# Comparative Mitogenome Analyses Uncover Mitogenome Features and Phylogenetic Implications of the Parrotfishes (Perciformes: Scaridae)

**DOI:** 10.3390/biology12030410

**Published:** 2023-03-07

**Authors:** Jiaxin Gao, Chunhou Li, Dan Yu, Teng Wang, Lin Lin, Yayuan Xiao, Peng Wu, Yong Liu

**Affiliations:** 1 Key Laboratory of South China Sea Fishery Resources Exploitation and Utilization, Ministry of Agriculture and Rural Affairs, South China Sea Fisheries Research Institute, Chinese Academy of Fishery Sciences, Guangzhou 510300, China; 2 Scientific Observation and Research Station of Xisha Island Reef Fishery Ecosystem of Hainan Province, Key Laboratory of Efficient Utilization and Processing of Marine Fishery Resources of Hainan Province, Sanya Tropical Fisheries Research Institute, Sanya 572018, China; 3 Guangdong Provincial Key Laboratory of Fishery Ecology Environment, Guangzhou 510300, China; 4 Observation and Research Station of Pearl River Estuary Ecosystem, Guangzhou 510300, China; 5 Key Laboratory of Aquatic Biodiversity and Conservation, Institute of Hydrobiology, Chinese Academy of Sciences, Wuhan 430072, China

**Keywords:** parrotfish, mitogenome, gene rearrangement, phylogeny, divergence time

## Abstract

**Simple Summary:**

Parrotfishes are among the most colorful and diverse inhabitants of the coral reefs and sea grass beds and are ecologically important in these habitats. Here, we presented the complete mitogenome sequences from twelve parrotfish species and conducted comparative analysis of mitogenome features among the seven published species for the first time. The comparative analysis revealed both the conserved and unique characteristics of parrotfish mitogenomes. The mitogenome structure, organization, gene overlaps, putative secondary structures of transfer RNAs, and codon usage were relatively conserved among all the analyzed species. However, the base composition and the intergenic spacers varied largely among species. All of the protein-coding genes were under purifying selection. Phylogenetic analysis revealed that the parrotfishes could be divided into two clades with distinct ecological adaptations. Early divergence of these two clades was probably related to the expansion of sea grass habitat, and later diversifications were likely associated with the geomorphology alternation since the closing of the Tethys Ocean. This work offered fundamental materials for further studies on the evolution and conservation of parrotfishes.

**Abstract:**

In order to investigate the molecular evolution of mitogenomes among the family Scaridae, the complete mitogenome sequences of twelve parrotfish species were determined and compared with those of seven other parrotfish species. The comparative analysis revealed that the general features and organization of the mitogenome were similar among the 19 parrotfish species. The base composition was similar among the parrotfishes, with the exception of the genus *Calotomus*, which exhibited an unusual negative AT skew in the whole mitogenome. The PCGs showed similar codon usage, and all of them underwent a strong purifying selection. The gene rearrangement typical of the parrotfishes was detected, with the *tRNA^Met^* inserted between the *tRNA^Ile^* and *tRNA^Gln^*, and the *tRNA^Gln^* was followed by a putative *tRNA^Met^* pseudogene. The parrotfish mitogenomes displayed conserved gene overlaps and secondary structure in most tRNA genes, while the non-coding intergenic spacers varied among species. Phylogenetic analysis based on the thirteen PCGs and two rRNAs strongly supported the hypothesis that the parrotfishes could be subdivided into two clades with distinct ecological adaptations. The early divergence of the sea grass and coral reef clades occurred in the late Oligocene, probably related to the expansion of sea grass habitat. Later diversification within the coral reef clade could be dated back to the Miocene, likely associated with the geomorphology alternation since the closing of the Tethys Ocean. This work provided fundamental molecular data that will be useful for species identification, conservation, and further studies on the evolution of parrotfishes.

## 1. Introduction

The mitochondrial genome (mitogenome) of a vertebrate is a small (16–17 kb), compact, and circular double-stranded molecule, typically encoding 13 protein-coding genes (PCGs), 22 transfer RNA genes (tRNAs), two ribosomal RNA genes (rRNAs), and two non-coding regions (the origin of L-strand replication, O_L_, and control region, CR) [1]. The mitochondrial DNA sequences have been extensively employed in a variety of study areas, from phylogeography, which elucidates the spatial arrangement of genetic variation among populations or closely related species [2,3,4], to phylogenetic studies, which decipher the evolutionary relationships across a wide range of taxa at higher taxonomic levels [5,6,7]. Compared to single or a few mitochondrial gene-based markers, the complete mitogenome sequences generally provide much finer phylogenetic resolution [8]. Moreover, genome-level characteristics, including nucleotide composition, genome structural arrangement, overlap, and non-coding intergenic spacers between genes, vary largely among different species and might possess evolutionary significance [9,10,11].

Parrotfishes (Scaridae) are among the most colorful and diverse inhabitants of coral reefs and sea grass beds [12]. Currently, a total of 100 species belonging to 10 genera are recognized, with *Scarus* being the most specious genus (52 species) [13]. These fish are mainly herbivorous, foraging mostly by excavating or scraping surfaces of rocks and carbonate substrate that are encrusted with algae, bacterial mats, and detritus [14]. As such, it is widely recognized that parrotfishes play an important role in marine bioerosion [15,16] and serve as determinants of benthic community structure [17]. For example, parrotfish can exert a top-down control on algal communities to provide more space and resources for coals and promote the attachment and recruitment of coral larvae [18,19,20]. Therefore, it can help to mitigate the competition between coral reefs and macroalgae and increase the resilience of coral reef ecosystems subjected to anthropogenic or natural disturbances [21,22]. In addition, the excavating and scraping species can break the reef framework into sand-sized sediments and facilitate the cycling of calcium carbonate on reefs, which are also dispensable agents in reef erosion and sediment production and transport [23,24].

Deciphering mitogenome structures and sequences can provide insights into evolutionary processes and contribute to species delimitation and conservation efforts [25,26]. Despite the fact that parrotfishes play an irreplaceable role in coral reef and sea grass bed habitats due to their unique behavioral and ecological characteristics, only a few studies have addressed their mitogenome characteristics [27,28,29], and comparative analysis is scarce. Although a handful of works have tried to elaborate on the phylogenetic relationships among the parrotfishes [30,31,32], none of them have addressed this question from a mitogenomic perspective. The deficiency of mitogenome data and comparative works hindered us from understanding the evolution of the parrotfish and establishing proper management and conservation decisions. In the present study, we reported twelve parrotfish mitogenomes for the first time and conducted comparative analysis with the published sequences from other seven species to elaborate the detailed features of the parrotfish mitogenomes. Additionally, we also investigated the phylogenetic relationships among these parrotfishes and estimated divergence times using mitogenome data. We hope that our newly generated data and results will provide some insights into the evolution of the parrotfishes as well as contributions towards the identification and conservation of these fishes.

## 2. Materials and Methods

### 2.1. Sampling, DNA Extraction, PCR Amplification, and Sequencing

In the present study, we de novo sequenced twelve parrotfish species (with one specimen each): Calotomus carolinus, Cetoscarus bicolor, Hipposcarus longiceps, Scarus globiceps, Scarus chameleon, Scarus rivulatus, Scarus dimidiatus, Scarus oviceps, Scarus frenatus, Scarus niger, Scarus prasiognathos, and Scarus quoyi. The specimens of parrotfish were obtained from the Xisha Islands (15°46′~17°08′ N, 111°11′~112°54′ E), China, and deposited in the South China Sea Fisheries Research Institute, Chinese Academy of Fishery Sciences. Thirteen published mitogenome sequences from seven parrotfish species (Bolbometopon muricatum, KY235362/NC033901; Calotomus japonicus, AP017568/NC035427; Chlorurus sordidus, AP006567; Scarus forsteni, FJ619271/NC011928; Scarus ghobban, FJ449707/NC011599; Scarus rubroviolaceus, FJ227899/NC011343; Scarus schlegeli, FJ595020/NC011936) were also included in the analysis.

Genomic DNA was extracted from either a small piece of flesh or a pelvic fin clip taken from the right side of the specimen using the E.Z.N.A.^®^ Tissue DNA Kit (OMEGA, Beijing, China) and following the manufacturer’s instructions. High-quality DNA samples were randomly broken into fragments with a length of 300~500 bp. Then complete genomic libraries were established using the Illumina TruSeq^TM^ Nano DNA Sample Prep Kit (Illumina, San Diego, CA, USA) following the manufacturer’s recommendation. The 150-bp paired-end sequencing was performed on the Illumina HiSeq2500 platform. Library construction and sequencing were performed by the Biozeron Corporation (Shanghai, China).

### 2.2. Sequence Assembly, Annotation, and Analyses

Prior to assembly, raw reads were filtered by Trimmomatic v0.39 [33] in order to remove the reads with adaptors, the reads showing a quality score below 20 (Q < 20), the reads containing a percentage of uncalled bases (“N” characters) equal to or greater than 10%, and the duplicated sequences. GetOrganelle 1.7.5 was used to assemble the mitogenomes [34]. The newly generated mitogenome sequences were deposited in Genbank under the accession numbers OQ349180-OQ349191. Annotation of the mitogenomes (PCGs, tRNAs, rRNAs, and CR) was performed using MITOS [35] and Mitoannotator v3.83 [36]. Transfer RNA (tRNA) genes and their secondary structures were determined by the MITOS webserver [35]. The base composition and codon distributions were analyzed in MEGA 7.0 [37], and the nucleotide composition skewness was calculated using the formulas (A − T)/(A + T) for AT skew and (G − C)/(G + C) for GC skew. Relative synonymous codon usage (RSCU) was calculated using DAMBE 7 [38]. The conserved sequence block domains (CSBs) were determined by comparing them with those of other species [1].

### 2.3. Phylogenetic Analyses

Prior to the phylogenetic analysis, the method of Xia et al. [39] was used to access substitution saturation of the sequences by comparing the information entropy-based index (*I_SS_*) with critical values (*I_SS.c_*) in DAMBE 7 [38]. If *I_SS_* is significantly lower than *I_SS.c_*, then sequences have not experienced substitution saturation. The sequence of the control region showed significant substitution saturation (*I_SS_* = 1.1897 > *I_SS.c_* = 0.7851, *p* < 0.001) and was thus excluded from further analysis. The phylogenetic relationships were reconstructed using the 13 PCGs and 2 rRNAs of the 19 parrotfish mitogenomes. Three *Cheilinus* species (*C. fasciatus*, NC037707; *C. oxycephalus*, NC061045; *C. undulatus*, NC013842) were used as outgroup taxa. Multiple sequence alignment was performed using MAFFT [40] implemented in PhyloSuite [41] under default parameters and subsequently checked by eye in SeaView [42]. Our dataset was partitioned by gene and codon position, and then the best-fit nucleotide substitution model for each partition was determined using Modelfinder [43]. Phylogenetic relationships were reconstructed using Bayesian inference (BI) and maximum likelihood (ML) approaches. BI was carried out in Mr. Bayes 3.2.7 [44]. Two independent Markov chains were run with 1 × 10^6^ iterations, and 10,000 trees were retained, with the first 25% of the samples discarded as burn-in. ML analysis was conducted in IQTREE [45] under 10,000 ultrafast bootstrap replicates. DNAsp 6 [46] was used to calculate non-synonymous substitution rates (dN), synonymous substitution rates (dS), and the ratio of dN/dS (ω).

MCMCTree, implemented in the PAML4.9i software package [47], was used to estimate the divergence time among the parrotfishes. The tree topology generated from BI was calibrated with fossil dates. The information of branch lengths, gradients, and hessian were first estimated with a maximum likelihood method in BsaeML of the PAML package. Then the MCMC approximation was performed with a burn-in period of 50,000 cycles, and a total of 10,000 samples were generated every 50 iterations. Two independent runs were performed. Tracer 1.7 [48] was used to check for effective sample sizes (ESS) of parameters. The ESS larger than 200 were considered to reach convergence.

Two fossil calibration points were used in the divergence time estimation. *Calotomus preisli* was known from the middle Miocene (~14 Ma) in Austria [49]. We calibrated the minimum age of the split between the sea grass clade and the coral reef clade using this fossil. The fossil elements belonging to the genus *Bolbometopon* were known from the late Miocene (~5.3 Ma) [49]. These fossils were used to set the minimum age of the separation between *Bolbometopon* and *Cetoscarus*. The root age of our phylogeny was set to be lower than 50 Ma, for the oldest known labrid fossil was dated back to 50 Ma from the Monte Bolca in Italy [50].

## 3. Results

### 3.1. General Features of Mitochondrial Genomes

**Figure 1:**
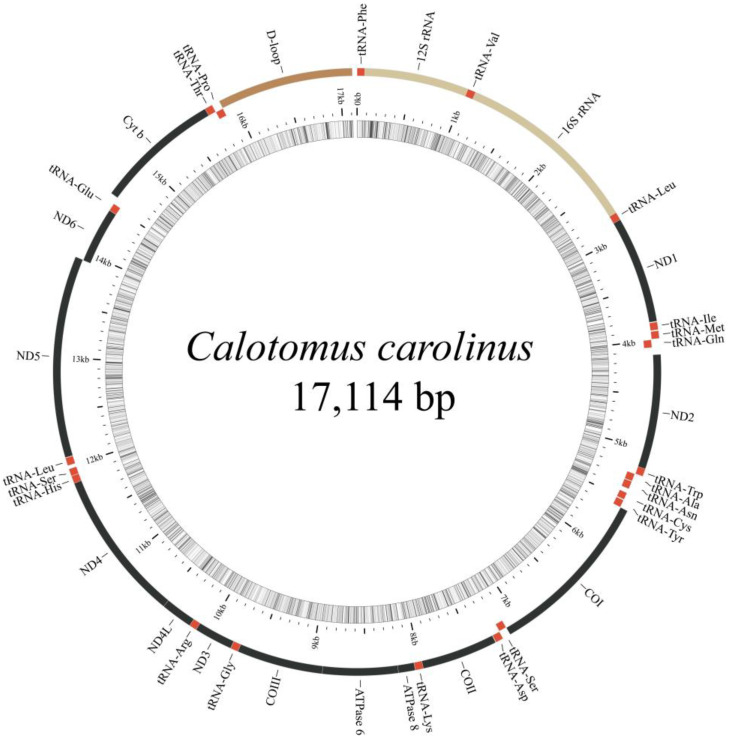
Organization of the parrotfish mitogenome. *Calotomus carolinus* was taken as an example. The inner ring indicated GC content.

The total length of the 12 newly sequenced complete mitogenomes ranged from 16,657 bp in *Scarus niger* to 16,816 bp in *Scarus globiceps*. The typical set of 37 genes, including 13 PCGs, two rRNAs, and 22 tRNAs, and a control region, were detected in all the mitogenomes (Table 1, Figure 1, Supplementary File S1: Table S1). All PCGs were encoded on the Heavy (H) strand except for NADH dehydrogenase subunit 6 (*ND6*), which was located on the Light (L) strand. Eight tRNAs (*tRNA^Gln^*, *tRNA^Ala^*, *tRNA^Asn^*, *tRNA^Cys^*, *tRNA^Tyr^*, *tRNA^Ser^* ^(UGA)^, *tRNA^Glu^* and *tRNA^Pro^*) were located on the L-strand, and the remaining 14 tRNAs were on the H-strand (Figure 1, Table 1). This coding pattern on the H and L-strand was identical among the 19 parrotfish species (Additional File 1: Table S1) and was consistent with most vertebrates [51].

### 3.2. Nucleotide Composition of the Parrotfish Mitogenomes and Unusual AT Skew of Calotomus Species

**Figure 2:**
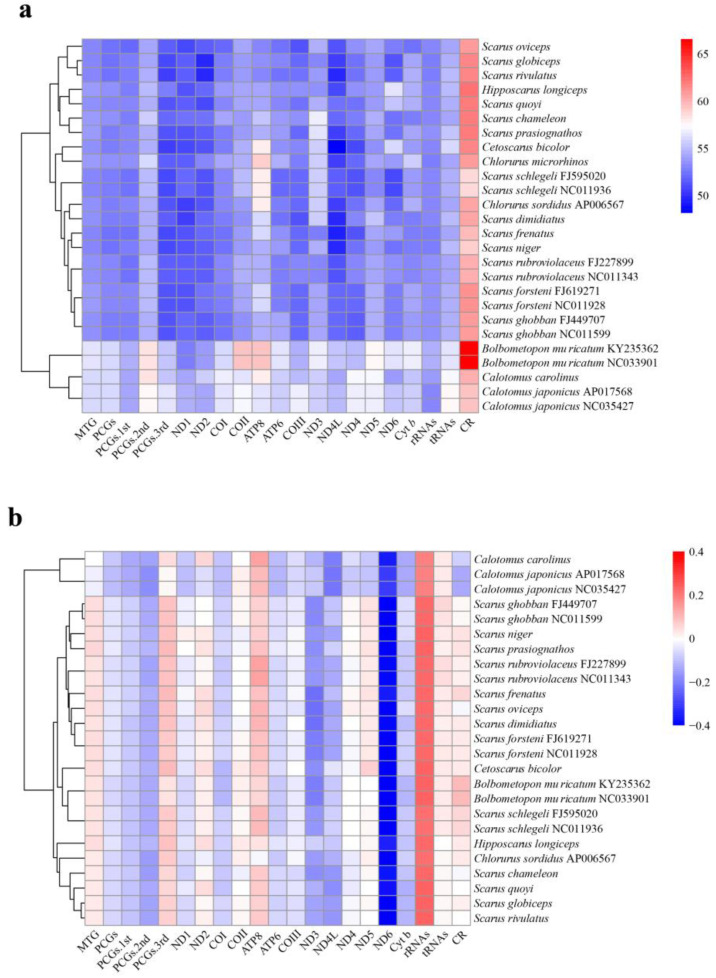
Base composition of various datasets among parrotfish mitogenomes, with hierarchical clustering of parrotfish species (y-axis) based on (**a**) AT content and (**b**) AT skew.

The nucleotide composition was similar among all of the parrotfish species, with the overall A + T content ranging from 53.0% in *Scarus globiceps* to 56.4% in *Bolbometopon muricatum*, and the A + T content was the lowest in *ND4L* (51.7 ± 3.6%) and the highest in the control region (62.6 ± 4.1%) (Figure 2a, Additional File 1: Table S2). All of the parrotfish mitogenomes exhibited AT bias, with the largest and most positive value observed in the rRNAs and the smallest and most negative value found in *ND6* (Figure 2b, Additional File 1: Table S3). Compared with other parrotfishes, species of the genus *Calotomus* exhibited an unusual AT skew for the whole mitogenomes with a slightly negative value (−0.04 to −0.02), while other species all displayed a positive value (Figure 2b, Additional File 1: Table S3). These results indicated that species of the genus *Calotomus* displayed an excess of T over A in the whole mitogenome.

### 3.3. Protein-Coding Genes

The total length of PCGs ranged from 11,391 bp to 11,415 bp, with *ATP8* being the shortest (168 bp) and *ND5* being the longest (1839 bp to 1848 bp). Most genes exhibited the typical start codon ATN. However, *COI* initiated with GTG in all species, and *ATP6* started with GTG in *Calotomus japonicus* and *Scarus oviceps* or CTG in *Calotomus carolinus* (Additional File 1: Table S1). Four types of stop codons were detected, including two canonical (TAA and TAG) and two truncated codons (T-- and TA-) (Additional File 1: Table S1). The incomplete stop codons were commonly observed in fish mitogenomes [1] and might be completed by post-transcriptional polyadenylation [52].

**Figure 3:**
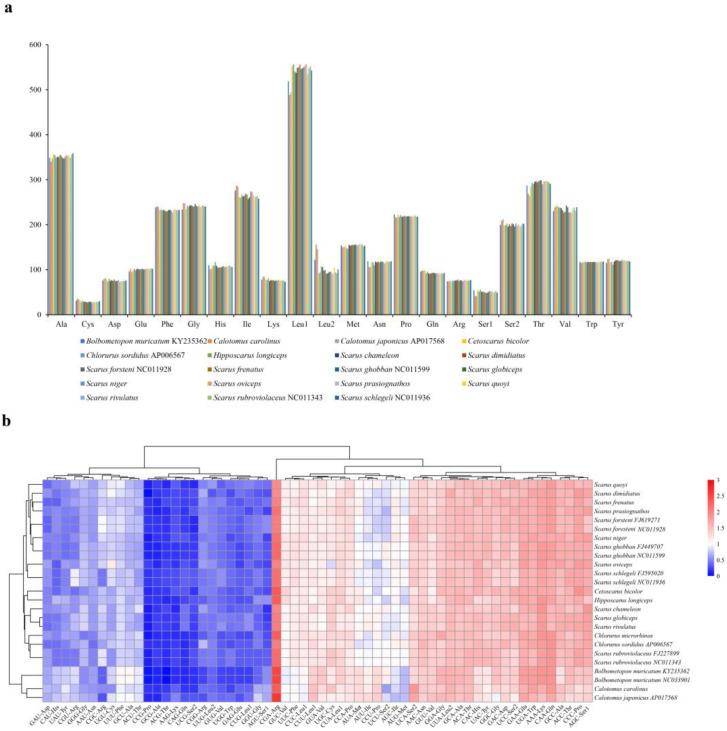
(**a**) Amino acid frequency in the parrotfish mitogenomes. (**b**) Heatmap based on the relative synonymous codon usage (RSCU) in the parrotfish mitogenomes.

For all the parrotfish mitogenomes, Leu^(CUN)^, Ala, and Thr were the three most frequently translated amnio acids, while Cys was the least used amnio acid (Figure 3a). Moreover, the most frequently used codon was CGA for arginine in all the parrotfish mitogenomes (Figure 3b). The RSCU revealed that degenerate codons were biased to use more A and T than G and C in the third codon position, which resulted in higher A + T content than G + C content in the third codon position of parrotfish mitogenomes (Figure 2a and Figure 3b, Supplementary File S2: Figure S1).

### 3.4. Gene Rearrangement and Secondary Structure of tRNAs

**Figure 4:**
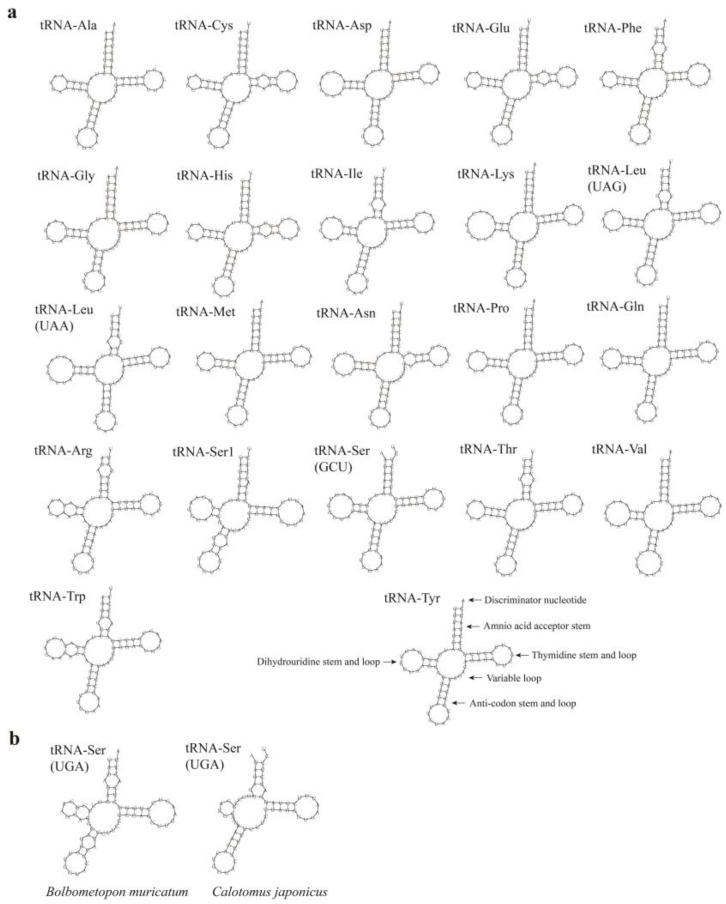
(**a**) Putative secondary structure of tRNAs in parrotfish mitogenomes. (**b**) Putative secondary structure of *tRNA^Ser^*^(AGN)^ in *Bolbometopon muricatum* and *Calotomus japonicus*.

All 22 tRNAs typical of the mitogenomes of vertebrates were found in the parrotfish mitogenomes (Figure 4a). Most tRNAs could be folded into the canonical clover-leaf secondary structure. The secondary structure of tRNAs generally consisted of four domains and a short variable loop: the amino acid acceptor (AA) stem, the dihydrouridine (D) arm (D stem and loop), the anticodon (AC) arm (AC stem and loop), the thymidine (T) arm (T stem and loop), and the variable (V) loop (Figure 4a). However, *tRNA^Ser^*^(AGN)^ in *Bolbometopon muricatum* and *Calotomus japonicus* possessed only small loop(s) in their D arms (Figure 4b), thus not forming the typical clover-leaf structure. A gene rearrangement of the tRNA gene cluster between *ND1* and *ND2* was detected, with the *tRNA^Met^* inserted between the *tRNA^Ile^* and *tRNA^Gln^*, and the *tRNA^Gln^* was followed by a putative *tRNA^Met^* pseudogene.

### 3.5. Overlaps and Non-Coding Intergenic Spacers

A total of four gene overlaps were detected in the mitogenome of *Calotomus carolinus* and five were observed in the mitogenomes of other parrotfishes (Table 1, Additional File 1: Table S1). The longest overlap was found between *ATP8* and *ATP6*, with highly conserved 10-bp motifs of “ATGGCACTAA” or “ATGACACTAA” detected in most parrotfish mitogenomes except for that of the genus *Calotomus*. The latter genus showed 16-bp overlaps of “CTGACCTTGGCACTAG” or “GTGGCCCTGGCACTAG”. Apart from that, a 7-bp overlap was observed between *ND4L* and *ND4* in all parrotfish mitogenomes with highly conserved sequences of “ATGCTAA” or “ATGTTAA”. 

**Figure 5:**
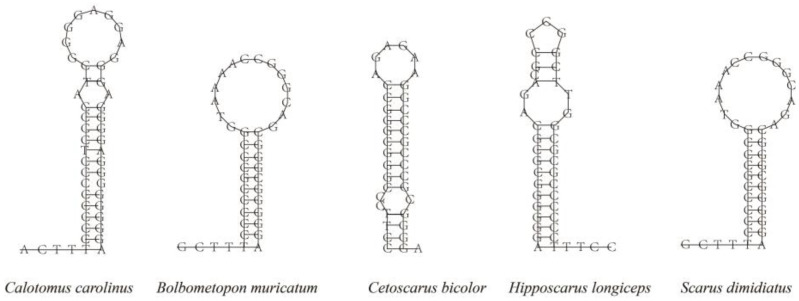
Putative secondary structure of the origin of L strand replication (O_L_) in five parrotfish species.

Two long intergenic spacers (IGS; *tRNA^Gln^*-*ND2* and O_L_) were found in all the parrotfish mitogenomes. Moreover, another long IGS between *tRNA^Glu^* and *Cyt b* was also found in the mitogenomes of the genus *Calotomus*. As mentioned above, the IGS between *tRNA^Gln^* and *ND2* was assumed to be a pseudogene of *tRNA^Met^*. O_L_ is located within the five tRNA gene cluster (WANCY), and its secondary structure showed a stable stem-loop hairpin, which is strengthened by 9 to 10 G-C base pairs (Figure 5). The G-C base pairs on the stem were highly conserved, while the loop varied in its base composition, with T being scarce. 

**Figure 6:**
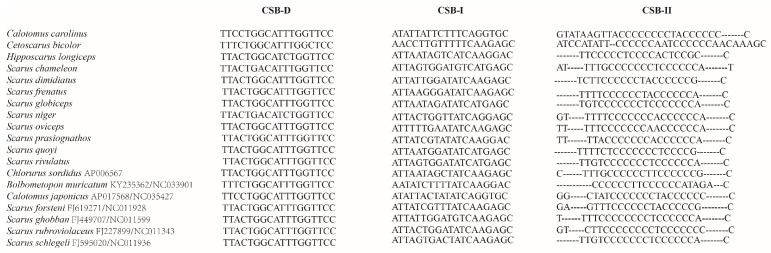
Conserved sequence blocks (CSBs) of the control region in the parrotfish mitogenomes.

The control region, located between *tRNA^Pro^* and *tRNA^Phe^*, was the most variable region and constituted the majority of the length variation of the parrotfish mitogenomes (Additional File 1: Table S1). Only three conserved sequence blocks (CSB-D, CSB-I, and CSB-II) were detected (Figure 6), with CSB-III completely missing in all the parrotfish mitogenomes. The base composition was extremely unique to each CSB, with CSB-D being T rich, CSB-I being AT rich, and CSB-II being C rich (Table 2).

### 3.6. Non-Synonymous and Synonymous Substitutions

**Figure 7:**
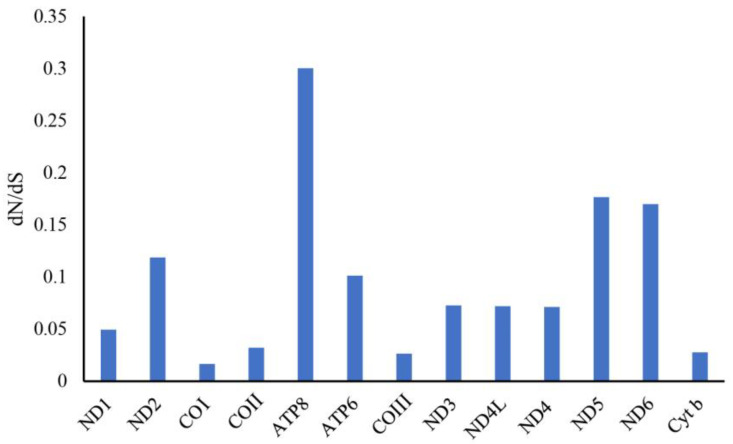
Non-synonymous/synonymous substitution ratios (ω) of the 13 PCGs in the parrotfish mitogenomes.

To better understand the role of selective pressure and the evolutionary patterns of the protein coding genes, the dN/dS value (ω) of each PCG was calculated (Figure 7). All of the PCGs were subject to purifying selection, with a dN/dS value lower than 1 (ω < 1). Among which, *ATP8* and *COI* presented the highest and lowest ω values (ω = 0.300 and 0.016), respectively.

### 3.7. Phylogenetic Relationships of the Parrotfishes

**Figure 8:**
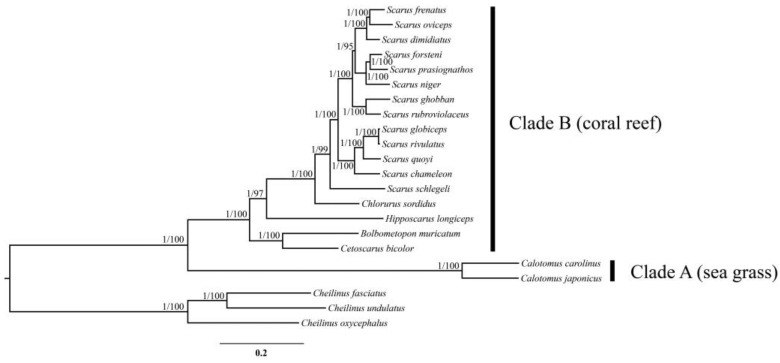
Phylogenetic relationships of the parrotfishes based on 13 PCGs and 2 rRNAs using Bayesian inference (BI) and maximum likelihood (ML). Numbers at nodes indicate Bayesian posterior probabilities (PP) and ultrafast bootstrap supports (UFBoot) from maximum likelihood analysis, respectively. Only well-supported numbers (PP > 0.95, UFBoot > 95) are shown.

The ML and BI trees based on the thirteen PCGs and two rRNAs yielded identical gene tree topologies (Figure 8), which congruently revealed two main clades. The first clade (clade A), located at the basal part of the tree, includes species of the genus *Calotomus*. The second clade (clade B) was comprised of the genera *Cetoscarus*, *Bolbometopon*, *Hipposcarus*, *Chlorurus*, and *Scarus*. The genus *Cetoscarus* was sister to *Bolbometopon*, positioned at the basal part of this clade. *Scarus* formed the sister genus to *Chlorurus*, then clustered with *Hipposcarus*. The monophyly of *Scarus* and *Chlorurus* was confirmed with strong support. Among the sampled *Scarus* species, *S. globiceps* showed a close relationship with *S. rivulatus* and exhibited little genetic difference (0.009 between the whole mitogenome). The nodes with high ML bootstrap support values and Bayesian posterior probabilities (BS > 70 and PP > 0.95) were shown.

### 3.8. Divergence Time Estimation

**Figure 9:**
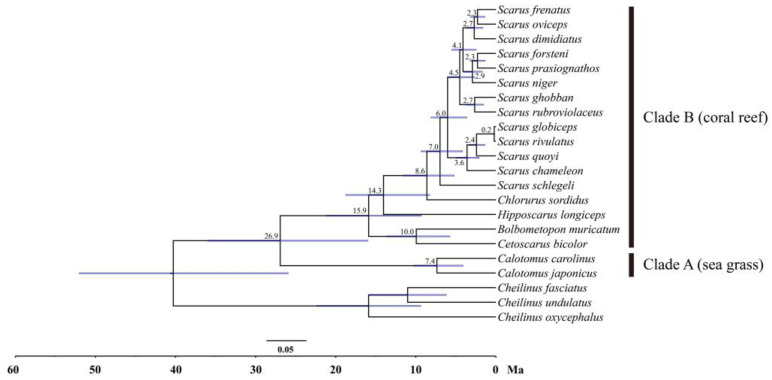
Divergence time estimation of the parrotfishes derived from MCMCTree analysis. Numbers at nodes indicate estimated age. Blue bars represent 95% credible age intervals for each node.

The estimated divergence time and 95% credible intervals (CIs) are shown in Figure 9. The split between clade A and clade B occurred at 26.9 Ma (95% CI 16.0~36.0 Ma) during the late Oligocene. The *Bolbometopon*-*Cetoscarus* clade differentiated at 15.9 Ma (95% CI 9.3~21.2 Ma) during the middle Miocene. *Hipposcarus* diverged at 14.3 Ma (95% CI 8.3~18.8 Ma). The split between *Chlorurus* and *Scarus* was dated back to 8.6 Ma (95% CI 5.2~11.6 Ma). The *Scarus* species diverged relatively recently, ranging from 0.2 Ma to 7.0 Ma.

## 4. Discussion

The comparative analysis revealed that the mitogenome structure, organization, codon usage, and putative secondary structures of tRNAs were highly similar among all the analyzed parrotfish species. The gene rearrangement of the tRNA gene cluster between *ND1* and *ND2* was detected, which is typical of parrotfish [27,28,29]. In parrotfish mitogenomes, the *tRNA^Met^* was located between the *tRNA^Ile^* and *tRNA^Gln^*, then a putative *tRNA^Met^* pseudogene was located after the *tRNA^Gln^*. The gene rearrangements had been proposed to occur with tandem duplication of gene regions as a consequence of slipped-strand mispairing, followed by deletions of redundant genes [53]. The *tRNA^Met^* pseudogene was believed to function as punctuation marks for mitochondrial *ND2* mRNA processing [27].

Previous studies on insects suggested that the intergenic spacers were important for transcription and might be associated with gene rearrangement [54,55,56]. Our results showed significant variance in IGS among the parrotfish mitogenomes, especially for the longest IGS, the control region. Despite the great length variations found in the CR of the parrotfish mitogenomes, three conserved sequence blocks could still be detected. Compared with most fish species [1], the CSB-III cannot be observed in the CR of the parrotfish mitogenomes. The lack of CSB-III was also reported in other vertebrates [57]. Up until now, the functions of the CSBs were still not clear, however, the common existence of CSB-D and CSB-I in vertebrate mitogenomes suggested that they were vital in the replication and transcription of the genome [1].

CR was commonly used as genetic markers in phylogenetic and population genetic analysis due to its high variability among populations and closely related species [58,59]. However, our analysis suggested that the CR of parrotfishes experienced significant substitution saturation. Substitution saturation reduces the amount of phylogenetic signals to the point that sequence similarities could probably be the consequence of chance alone rather than homology. Therefore, phylogenetic signals are lost, and the sequences are no longer informative about the underlaying evolutionary processes that generate them if substitution saturation is reached [60]. For example, the mitochondrial markers *COI* and *ND3* that are commonly used in phylogenetic studies and DNA barcoding were proven to be subjected to significant substitution saturation in Caryophyllidean cestodes. Therefore, arbitrary application of these markers to the phylogenetic inference of this group of cestodes would jeopardize the well-supported phylogenetic estimates and evolutionary relationships [61]. In our case, the CR sequences have never been employed to infer the phylogenetic relationships among parrotfishes so far [30,31,32]. Future studies should avoid using the CR sequences when it comes to phylogenetic relationship inference or identification via DNA barcoding of the parrotfishes. 

The RSCU revealed that degenerate codons were prone to use more A and T than G and C in the third codon position, therefore higher A + T content than G + C content was observed in the third codon position of parrotfish mitogenomes (Figure 2a and Figure 3b, Additional File 2: Figure S1). This phenomenon was frequently observed in other teleosts [1] and might be related to genome bias, optimal selection of tRNA, or DNA repair efficiency [62].

Compared with other parrotfish species, the *Calotomus* species displayed an unusual AT skew for the whole mitogenome with a slightly negative skewness, while other species all showed positive values. Nucleotide skewness might be related to the balance between mutational and selective pressures during replication [63,64,65]. Some previous studies had indicated that the preference for certain nucleotides might be associated with selection rather than mutation [66]. For example, *Sinorhodeus microlepis*, a bitterling species with highly specialized ecological and behavioral preferences [67], also exhibited an unusual AT skew in its mitogenome [68] and this unique AT skewness was believed to be associated with unique selective forces [68]. Compared with other parrotfish species, the *Calotomus* species possessed some unique ecological aspects, such as the browsing feeding behavior and the lack of breeding territories [30]. It is suspected that distinct selective pressures or processes might lead to the preference of T in their mitogenomes. However, what and how the selective processes account for the unusual AT skew in the *Calotomus* species needs further investigation.

All of the PCGs were evolved under the purifying selection (ω < 1). The lower ω value on the whole suggested a prevalent signature of strong functional restrictions across the mitogenome, which was largely in agreement with the functional importance of mitochondria as a respiration chain necessary for OXPHOS and electron transport [69]. Furthermore, the lower ω value indicated fewer variations in the amino acids; therefore, *COI* and *Cyt b* could serve as potential barcoding markers for the identification of parrotfish.

The phylogenetic relationships among the parrotfish genus based on thirteen PCGs and two rRNAs of the mitogenome indicated two distinct clades (A and B), which were identical with previous studies based on concatenated data of both mitochondrial and nuclear markers [30,31,32]. These two clades recovered by the phylogenetic analysis correspond to two distinct groups with different aspects of ecological adaptation, which had been defined as the sea grass clade and the coral reef clade [30]. The sea grass clade, as represented by *Calotomus* in this study, exhibited some less modified morphological characteristics (e.g., discrete teeth without cementation) [70] and showed some distinct ecological and behavioral aspects (e.g., browsing, no breeding territories, and no harem) [30]. These features differed greatly from the coral reef clades. In addition to the phylogenetic analysis, our results also revealed some unique features of the mitogenome composition and organization in the *Calotomus* mitogenomes (e.g., unusual AT skewness in the mitogenome and additional IGSs), indicating the evolutionary distinctiveness of the sea grass clade. 

The first split of the parrotfish was estimated to have occurred in the late Oligocene (26.9 Ma, 95% CI 16.0~36.0 Ma), separating the sea grass clade from the coral reef clade. Geological evidence suggests that tectonic movements in the Indo-West Pacific region during the late Oligocene and early Miocene resulted in the formation of vast areas of shallow-water habitat between Australia and Indonesia [71], facilitating the expansion of sea grass habitat [72]. Our divergence time estimation was largely in congruence with the timing of the large-scale development of the sea grass habitat. This result probably indicated that the ecological differences between these two habitats acted as the major driving force in the early diversification of the parrotfishes. The differentiation within the coral reef clade had been initiated since the middle Miocene (about 15.9 Ma), which is well consistent with the closure of the Tethys Ocean [73]. Alterations in geomorphologies such as sea levels, sea surface temperatures, and ocean circulations exerted a great impact on coral reefs [74,75,76], likely functioning as the driving forces behind the rapid radiation of coral reef species. Previous studies indicated that the extensive diversification of coral reef taxa occurred during this period and was likely associated with the geomorphological reconfiguration of the marine realm [77]. In addition, natural and sexual selections might have also contributed to the diversification of parrotfishes. Some studies suggested that the protogynous mating system of parrotfishes might function as a possible driving force of speciation [30]. Though some research has suggested that ecological and selection may operate in tandem in the speciation processes [31], the function mechanisms and their relative roles still require further investigation.

## 5. Conclusions

In the present study, comparative analysis revealed both the conserved and unique characteristics of parrotfish mitogenomes. The mitogenome structure, organization, gene overlaps, putative secondary structures of tRNAs, and codon usage were relatively conserved among all the analyzed species. However, the base composition and the intergenic spacers varied largely among species. All of the PCGs were under purifying selection. Phylogenetic analysis revealed that the parrotfishes could be divided into two clades with distinct ecological adaptations. Early divergence of the sea grass and coral reef clades occurred in the late Oligocene, probably related to the expansion of sea grass habitat. Later diversification within the coral reef clade could be dated back to the Miocene, likely associated with the geomorphology alternation since the closing of the Tethys Ocean. This study offered fundamental molecular materials for further studies on the evolution and diversification of the parrotfishes and would contribute to their identification and conservation.

## Figures and Tables

**Table 1:** Features of the mitochondrial genome of the parrotfishes. *Calotomus carolinus* was taken as an example.

Features	Start	Stop	Length/bp	Intergenic Nucleotide	Start Codon	Stop Codon	Anti-Codon	Strand
*tRNA^Phe^*	1	69	69	0			GAA	+ *
*12S-rRNA*	70	1020	951	0				+
*tRNA^Val^*	1021	1093	73	0			TAC	+
*16S-rRNA*	1094	2782	1689	0				+
*tRNA^Leu^* ^(UAA)^	2783	2855	73	0			TAA	+
*ND1*	2856	3830	975	7	ATG	TAA		+
*tRNA^Ile^*	3838	3907	70	10			GAT	+
*tRNA^Met^*	3918	3986	69	6			TTG	+
*tRNA^Gln^*	3993	4063	71	68			CAT	−
*ND2*	4132	5176	1045	0	ATG	TAG		+
*tRNA^Trp^*	5177	5247	71	4			TCA	+
*tRNA^Ala^*	5252	5322	71	5			TGC	−
*tRNA^Asn^*	5328	5400	73	41			GTT	−
*tRNA^Cys^*	5442	5507	66	9			GCA	−
*tRNA^Tyr^*	5517	5586	70	1			GTA	−
*COI*	5588	7138	1551	0	GTG	TAA		+
*tRNA^Ser (UGA)^*	7139	7209	71	3			TGA	−
*tRNA^Asp^*	7213	7283	71	4			GTC	+
*COII*	7288	7978	691	0	ATG	T		+
*tRNA^Lys^*	7979	8052	74	1			TTT	+
*ATPase 8*	8054	8221	168	−16	ATG	TAG		+
*ATPase 6*	8206	8894	689	0	CTG	TA		+
*COIII*	8895	9679	785	0	ATG	TAA		+
*tRNA^Gly^*	9680	9750	71	1			TCC	+
*ND3*	9752	10103	352	0	ATA	TAG		+
*tRNA^Arg^*	10,104	10,172	69	0			TCG	+
*ND4L*	10,173	10,469	297	−7	ATG	TAA		+
*ND4*	10,463	11,843	1381	0	ATG	T		+
*tRNA^His^*	11,844	11,912	69	2			GTG	+
*tRNA^Ser (GCU)^*	11,915	11,980	66	32			GCT	+
*tRNA^Leu (UAG)^*	12,013	12,084	72	4			TAG	+
*ND5*	12,089	13,930	1842	−4	ATG	TAA		+
*ND6*	13,927	14,448	522	1	ATG	TAA		−
*tRNA^Glu^*	14,450	14,522	73	64			TTC	−
*Cyt b*	14,587	15,727	1141	0	ATG	T		+
*tRNA^Thr^*	15,728	15,799	72	0			TGT	+
*tRNA^Pro^*	15,800	15,872	73	0			TGG	−
*D-loop*	15,873	17,114	1242					+

* +/− indicated H strand and L strand, respectively; a negative value indicated overlapping nucleotides.

**Table 2:** Base composition of the CSBs of parrotfish mitogenomes.

Base Composition (%)	CSB-D	CSB-I	CSB-II
A	10.5	34.2	8.8
T	44.4	32.2	20.1
G	21.6	19.6	5.2
C	23.5	14.0	65.9

## Data Availability

The data presented in this study are available in NCBI GenBank (Accession number: OQ349180-OQ349191).

## Supplementary Materials

The following supporting information can be downloaded at: https://www.mdpi.com/article/10.3390/biology12030410/s1, Table S1: General features of the parrotfish mitogenomes; Table S2: AT content of the parrotfish mitogenomes; Table S3: AT skew of the parrotfish mitogenomes; Figure S1: Relative synonymous codon usage (RSCU) of the 12 newly determined parrotfish species.
