# Supplementary figures and images for: Comparative Mitogenome Analyses Uncover Mitogenome Features and Phylogenetic Implications of the Parrotfishes (Perciformes: Scaridae)
Source: Biology (Basel). 2023 Mar 7;12(3):410. doi: 10.3390/biology12030410 (PMC10044791; doi:10.3390/biology12030410)

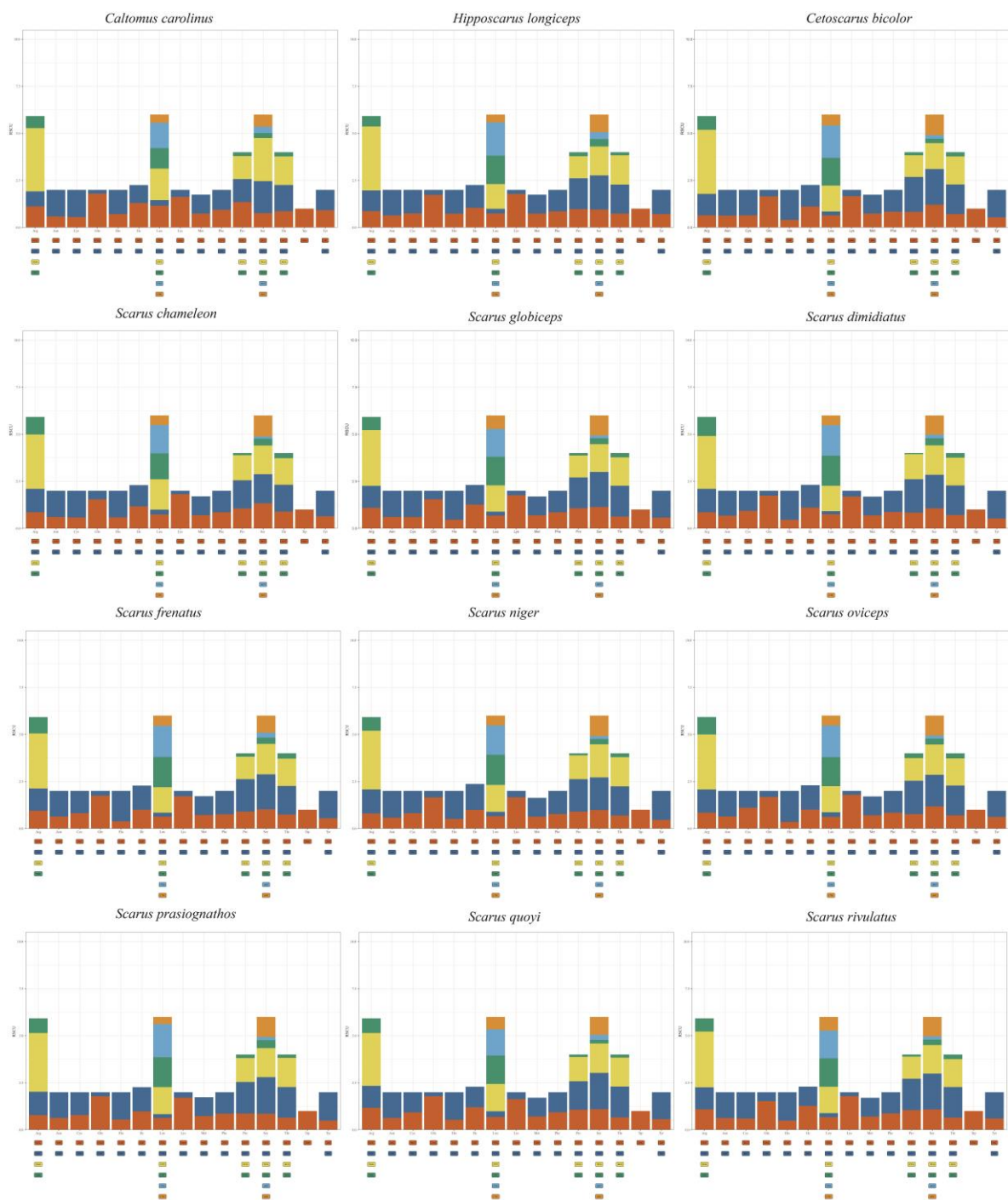

**Figure S1.** Relative synonymous codon usage (RSCU) of the 12 newly determined parrotfish species.

Supplement: Supplementary file 1 [file biology-12-00410-s001.zip › biology-2221904-Figure S1.pdf]
